# Bispectral Index Changes during Acute Brainstem TIA/Ischemia

**DOI:** 10.1155/2010/697185

**Published:** 2010-06-03

**Authors:** Chris P. Bleeker, Bart Smits, Pieter E. Vos, Jo M. J. Mourisse

**Affiliations:** ^1^Department of Anesthesiology, Radboud University Nijmegen Medical Centre, P.O. Box 9101, 6500 HB Nijmegen, The Netherlands; ^2^Department of Neurology, Radboud University Nijmegen Medical Centre, P.O. Box 9101, 6500 HB Nijmegen, The Netherlands

## Abstract

We describe a 76-year-old patient who suffered a brainstem TIA just before being anesthetised for cardiac surgery. The TIA was registered on BIS and resulted in a drop in BIS to a value of 60. When consciousness returned spontaneously, the BIS increased to 85. The relative use of the BIS during an operation is discussed. We believe that the lack of input from the brainstem to the frontal cortex resulted in the reduced cortical electrical activity as registered with the BIS.

## 1. Introduction

In cardiac anesthesia BIS monitoring is increasingly used to monitor anesthesia depth as well monitoring cerebral ischemia, which may be particularly important during cardiopulmonary bypass (CPB) [1, 2] and cardiopulmonary resuscitation [3, 4]. We describe a patient who, while being monitored with BIS, suffered a transient ischemic attack (TIA) of the brainstem.

## 2. Case Report

A 76-year-old man was scheduled for a three-vessel coronary artery grafting (CAG) using CPB.

His medical history included hypertension, a minor inferior myocardial infarction, amaurosis fugax, surgical resection of a vocal cord carcinoma, and an eyelid correction.

Physical examination showed a 70 kg, 1.69 m tall patient with a blood pressure of 170/70 mmHg and a pulse of 52. A bruit was heard over the heart, both carotid arteries and femoral arteries consistent with an aortic valve sclerosis. Cardiac ultrasound showed a hypokinetic inferior left ventricular wall, a good left and right ventricular function and an aortic valve sclerosis with minor aortic valve insufficiency. Coronary angiogram showed occlusion of the RCA, 90% occlusion of the LAD and 70% occlusion of the RCX. Patient took acetylsalicylic acid, chlortalidone, amlodipine, atorvastatin, metoprolol, isosorbide mononitrate, and isosorbide dinitrate. The patient was scheduled for a coronary bypass and premedicated with paracetamol 1000 mg and midazolam 7.5 mg, orally. Once in the operating theatre the patient was prepared for the operation with an 14 G IV infusion and a 20 G arterial cannula in the left radial artery as well as standard monitoring with a 5 lead ECG, pulsoximetry, and a noninvasive blood pressure band on the right arm. A BIS Quatro Sensor (XP) was placed and the monitor (Aspect Medical Systems, Inc. model A-2000 BIS Monitor) started. During this period the patient was alert and communicative. Without provocation, the patient suddenly complained of dizziness, stopped breathing, became unresponsive, and his eyes deviated upwards en laterally. The BIS monitor then gave its first reading of 60. 

We initially ventilated the patient by mask and gave naloxone to rule out an accidental sufentanil bolus. Subsequently the patient was intubated without medication. We requested an emergency neurological consultation. During this period the blood pressure and pulse remained stable at around 170/85 with a pulse of 55.

Neurological examination showed a Glasgow Coma Score (GCS) of E-1, M-1, and V-T: eyes closed, no motor response to painful stimuli, and no sounds. He had pinpoint pupils and pupillary reactions showed minimal constriction to light. Corneal reflexes were present while oculocephalic reflexes were absent. Both eyes were deviated upwards and to the left. Tendon reflexes were increased on the left side, with bilateral extensor plantar responses. 

We performed a CT-scan, which showed central and cortical atrophy with subthalamic and periventricular hypodensities consistent with older vascular damage. The CT-angiogram showed generalized atherosclerotic changes in all brachiocephalic vessels, especially in the common and internal carotid arteries and a slight stenosis of the origin of the left vertebral artery. 

After the CT-scan, we restarted the BIS monitoring which still showed the BIS value at about 50 to 60. Eleven minutes after resuming BIS monitoring the patient opened his eyes, started breathing and responding to voice commands, and BIS values increased to around 80–85. The patient was extubated and wanted to know the result of the operation. On neurological examination, the patient was alert and responded adequately. There was a bilateral downbeat nystagmus in downgaze. Tendon reflexes remained increased on the left side, while both plantar responses were flexor. Based on the neurological examination, the transient nature of the neurological deficit, and the CT-scan, we concluded that the patient had suffered a transient ischemic attack of the brainstem.

During the whole event which lasted 102 minutes the patient had remained hemodynamically stable. He was transferred to the neurological intensive care where the rest of his stay remained unremarkable, and he recovered without any neurological deficit.

## 3. Discussion

The primary reason to use an EEG monitor in anesthesia is to prevent awareness. Indeed, individuals suffering from this experience can have serious mental disorders. Awareness in a general population was found to be just below 0,2% and in a high-risk population just below 1% [5, 6]. Two large scale prospective trials (SAFE trial and B-Aware trial [5, 6]) demonstrated a reduction of 80% in the incidence of awareness using the BIS monitor. Furthermore, BIS and other EEG devices can be used to titrate anesthetics towards a desired level of hypnosis, with the aim to prevent exaggerated plasma concentration, which could lead to hemodynamic instability and prolonged awakening.

There are some clinical conditions where BIS is unusually low. These are conditions were the cerebral functions are impaired by hypoperfusion, ischemia, hypoglycaemia, and hypothermia [7]. Patients with a neurological disease can present with unusual BIS values and anticonvulsant drugs may reduce BIS values. BIS may also detect microembolic injury [8]. 

In our case, BIS monitoring coincided with the clinical findings of reduced cerebral activity and later the return of consciousness. The BIS value is derived from two frontal leads. As such it will primarily monitor frontal lobe cortical electrical activity and indirectly frontal cerebral perfusion. As there was no change in blood pressure and pulse rate we think that the overall cerebral perfusion pressure was not reduced during the ischemic attack. Therefore, together with the diagnosis of brainstem TIA, we think that the BIS value is decreased by another mechanism then a perfusion disturbance. The cerebral cortex receives extensive afferent projections from brainstem nuclei. There is a long list of pathological brain conditions known to affect the EEG. Ischaemia is one of these conditions. Ischaemia of the lower brainstem (with a clinical picture characterized by coma, respiratory abnormalities, and pinpoint pupils) results in diffuse low-voltage activity and bilateral slowing of the EEG although a posterior alpha rhythm may be preserved. Hence a logical explanation for the significant decrease in the BIS signal observed in our patient may be the decrease in frontal cortical activity during ischemia of the brainstem. However, people using the BIS should be aware that the simplifying algorithm built in to the monitor limits its diagnostic possibilities and there are many factors, unknown to the user, which can alter the BIS value. The BIS monitoring did not lead to a change in treatment policy.

Brainstem TIAs are supposedly a rare occurrence. The timing of our patients attack was quite spectacular: 20 minutes earlier and he would have been found asphyxiated in his bed and his death would most likely have been attributed to a coronary event. Thirty seconds later he would have been under anesthesia and subjected to hypothermia, hypotension, and full heparinization from the CPB. We can only speculate what the neurological outcome would have been. If the neurological outcome had been bad, it would have been labeled as a complication of the CPB. It begs the question how often the brainstem TIAs really happen in our cardio vascular-compromised population.

## 4. Summary

This 76-year-old patient suffered a brainstem TIA before cardiac surgery. The TIA was registered on BIS and resulted in a drop in BIS to a value of 60. When consciousness returned spontaneously, the BIS increased to 85. We believe that the lack of input from the brainstem to the frontal cortex resulted in the reduced cortical electrical activity as registered with the BIS.
